# Clinical efficacy of norepinephrine combined with cimetidine in treatment of neonatal upper gastrointestinal hemorrhage and its adverse reactions

**DOI:** 10.12669/pjms.38.8.5732

**Published:** 2022

**Authors:** Xuyan Dong, Hongjun Li, Tianjiao Zhu

**Affiliations:** 1 Xuyan Dong, Department of Obstetrics, Huangshi Maternity & Children’s Health Hospital, Edong Healthcare Group, Women’s & Children’s Hospital Affiliated to Hubei Polytechnic University, Xianning 435003, Hubei, P.R. China; 2 Hongjun Li, Department of Obstetrics, Xiaogan xiaonan district maternal and child care service center, Xiaogan 432100, Hubei, P.R. China; 3 Tianjiao Zhu Department of Obstetrics, Xianning Central Hospital, The First Affiliated Hospital of Hubei University of Science and Technology, Xianning 431000, Hubei, P.R. China

**Keywords:** Neonatal upper gastrointestinal hemorrhage, Norepinephrine, Cimetidine, Adverse reaction

## Abstract

**Objectives:**

To investigate the clinical efficacy of norepinephrine combined with cimetidine in the treatment of neonatal upper gastrointestinal hemorrhage and its adverse reactions.

**Methods:**

A total of 68 cases of neonatal upper gastrointestinal hemorrhage in Huangshi Maternal and Child Health Care Hospital from please mention dates October 2018 to February 2020 were selected and randomly divided into treatment group and control group by coin tossing, with 34 infants in each group. The control group received conventional therapy, and the treatment group was additionally treated with norepinephrine combined with cimetidine. The efficacy and safety were compared between the two groups.

**Results:**

The time when the bleeding stops, the time of fecal occult blood turning negative and hospital stay of the treatment group were shorter than those of the control group (*P* < 0.05). Superoxide dismutase (SOD) level increased while malondialdehyde (MDA) level decreased in both groups after treatment compared with those before treatment (*P* < 0.05). After treatment, the SOD level was higher while the MDA level was lower in the treatment group than those in the control group (*P* < 0.05). The effective rate of the treatment group was higher than that of the control group (*P* < 0.05). However, no significance was found in adverse reactions between the two groups (*P* > 0.05).

**Conclusion:**

Norepinephrine combined with cimetidine in the treatment of neonatal upper gastrointestinal hemorrhage can shorten the recovery time of symptoms, improve efficacy and reduce stress reaction. It is safe, effective and worthy of use in clinical practice.

## INTRODUCTION

Neonatal upper gastrointestinal hemorrhage is a common acute and critical neonatal disease, with high-risk factors including asphyxia, premature delivery, low body weight, infection, premature weaning, gastrointestinal lesions and systemic lesions. Affected by bleeding site and volume, its early clinical manifestations are commonly vomiting, abdominal distension, bloody stool, shock and intra-abdominal free gas, which are prone to threaten the life health of infants without timely intervention or treatment.^1^,^2^ At present, the main clinical treatment for neonatal upper gastrointestinal hemorrhage is symptomatic hemostasis, that is, fasting, gastric lavage with sodium chloride solution, thrombin, vitamin K1 and reptilase, as well as appropriate oxygen therapy and blood transfusion for patients with massive hemorrhage.^3^ The value of improving infants’ symptoms through early hemostasis has been widely affirmed.^4^ Moreover, it has been recently found that cimetidine and norepinephrine has a good effect in upper gastrointestinal hemorrhage, which has been rarely reported in neonatal upper gastrointestinal hemorrhage.^5^-^7^ On this basis, in our study, 68 cases of neonatal upper gastrointestinal hemorrhage were selected to analyze the effect and safety of their combined use.

## METHODS

The infants with neonatal upper gastrointestinal hemorrhage treated in Huangshi Maternal and Child Health Care Hospital from October 2018 to February 2020 were randomly divided into treatment group and control group, with 34 infants in each group. The control group included 19 males and 15 females, aged between 1-22 days (Mean, 11.57 ± 2.39 days). Among them, there were 26 term infants and 8 premature infants. There were 20 cases of neonatal asphyxia (59%), 9 cases of neonatal pneumonia (26%), 2 cases of septicemia (6%), and 3 cases of others (9%). In the treatment group,there were 18 males and 16 females, with an age of 1-21 days (Mean, 11.43 ± 2.28 days), of which 28 were term infants and 6 were premature infants. The type of primary diseases included neonatal asphyxia (n = 21, 62%), neonatal pneumonia (n = 10, 29%), septicemia (n = 2, 6%) and others (n = 1, 3%). The general data showed no significant differences between the two groups (*P* > 0.05).

### Ethical Approval:

The study was approved by the Institutional Ethics Committee of Huangshi Maternity & Children’s Health Hospital on May 2020 (No.[2020]033), and written informed consent was obtained from all participants

### Inclusion criteria:

- Family members signing the informed consent;
- Infants meeting the clinical diagnostic criteria for neonatal upper gastrointestinal hemorrhage; - Infants without drug allergy
- Infants with occult blood test-positive in gastric contents and feces.

### Exclusion criteria:

- Patients with immune deficiency,
- Heart disease or other serious organic impairment;
- Patients with coagulopathy;
- Patients with digestive tract malformation;
- Patients with systemic hemorrhagic disease or swallowing syndrome;
- Patients with an allergic constitution;
- Patients whose family members refused to participate in the study or transferred to another hospital.

After the infants were admitted, their families were educated by professional nursing staff, and the infants were placed in the supine position keeping them warm. The control group was given conventional treatment, including gastric lavage, fasting, intravenous nutrition, correction of water-electrolyte and acid-base-equilibrium disorders, 0.5-1 mg/(kg·d) vitamin K1 injection (Chinese medicine Zhunzi H34021789, produced by Wuhu Kangqi Pharmaceuticals Co., Ltd.), and 25 mg/(kg·d) ethamsylate injection (Chinese medicine Zhunzi H42020039, produced by Hubei Tianyao Pharmaceutical Co., Ltd.). The infected children were treated with antibiotics according to the drug sensitivity results. On this basis, the treatment group was additionally treated with norepinephrine combined with cimetidine by injecting 8 mg noradrenaline bitartrate injection (Chinese medicine Zhunzi H42021301, produced by Grandpharma (China) Co., Ltd.) and 100 ml 0.9% cold sodium chloride solution through a nasogastric tube, 10-20 ml/time. The tube was preserved for 30 minutes and then withdrawn. Infants with severe bleeding could be injected once every 2-4 hour, and the medication time could be delayed gradually after the symptoms were relieved. Subsequently, 10-15 mg/(kg·d) cimetidine injection (Chinese medicine Zhunzi H44024630, produced by Guangdong Nanguo Pharmaceutical Co., Ltd.) mixed with 20 ml 0.9% sodium chloride injection was given intravenously 1-2 times/day. After hemostasis is achieved, the intravenous drip was continued for 2-3 days.

### Observation Indicators:

The time when the bleeding stops, the time of fecal occult blood turning negative and the average length of hospital stay were observed. Venous blood (2 ml) was collected from the infants before and after treatment. After serum separation, the supernatant was collected to measure superoxide dismutase (SOD) and malondialdehyde (MDA) in plasma using the hydroxylamine method and thiobarbituric acid method, respectively. Evaluation of clinical treatment effect: according to the actual treatment effect of the two groups were remarkably effective, remission and ineffective respectively. Remarkably effective: upper gastrointestinal bleeding symptoms were significantly improved, bleeding stopped within 24 hours, no serious bleeding tendency. Remission: upper gastrointestinal bleeding symptoms were improved to a certain extent, bleeding stopped 24-72 hours, bleeding tendency was general. Ineffective: the symptoms of upper gastrointestinal bleeding did not improve significantly, and the bleeding did not stop over 72 h, resulting in severe bleeding trend. Total effective rate = (remarkably effective cases + remission cases)/total cases * 100.00%. The adverse reactions of the two groups were recorded during treatment.

### Statistical Analysis:

The results were analyzed using SPSS 22.0. The measurement data were expressed as(x̄ ±s) and analyzed with the t test. The enumeration data was expressed as % and analyzed using the X^2^ test. *P* < 0.05 was considered as statistically significant.

## RESULTS

The hemostasis time, time of occult blood turning negative and hospital stay of the treatment group were shorter than those of the control group (*P* < 0.05). Table-I. Before treatment, there was no significant difference in SOD and MDA levels between the two groups, which was comparable (*P* > 0.05). After treatment, SOD level in the two groups increased, and SOD level in the treatment group was significantly higher than that in the control group (*P* < 0.05). The level of MDA in both groups decreased after treatment, and the level of MDA in treatment group was lower than that in control group (*P* < 0.05). This suggested that the peroxidation reaction in the children was significantly inhibited after treatment, and the inhibition effect was more obvious in the treatment group.

**Table-I:** Comparison of relevant indicators (x̄± s).

Group	n	Hemostasis time (h)	Time of occult blood turning negative (h)	Average length of hospital stay (d)
Treatment group	34	15.34 ± 4.58	30.26 ± 7.54	3.54 ± 1.03
Control group	34	23.14 ± 5.92	48.94 ± 8.17	6.74 ± 2.15
t	-	6.077	9.797	7.827
P	-	0.000	0.000	0.000

### Efficacy Analysis:

The efficacy of the treatment group was higher than that of the control group (*P* < 0.05).Table-III.

**Table-II:** Comparison of stress indicators (x̄±s).

Group	n	SOD (U/ml)		MDA (μmol/L)	

		Before treatment	After treatment	Before treatment	After treatment
Treatment group	34	26.54 ± 7.54	41.57 ± 8.92	58.91 ± 10.24	30.26 ± 7.57
Control group	34	26.61 ± 7.16	32.71 ± 7.64	58.78 ± 10.07	44.18 ± 8.91
t	-	0.039	4.399	0.053	6.942
P	-	0.969	0.000	0.958	0.000

**Table-III:** Comparison of efficacy [n (%)]

Group	n	Remarkably effective	Remission	Ineffective	Effective rate
Treatment group	34	20 (58.82)	13 (38.24)	1 (2.94)	33 (97.06)
Control group	34	12 (35.29)	15 (44.12)	7 (20.59)	27 (79.41)
x^2^	-				5.100
P	-				0.024

### Analysis of adverse Reactions:

No statistically significant differences were found in adverse reactions between the two groups. Table-IV.

**Table-IV:** Comparison of adverse reactions [n (%)].

Group	n	Allergy	Rashes	Vomiting	Constipation	Total incidence
Treatment group	34	0 (0.00)	0 (0.00)	1 (2.94)	0 (0.00)	1 (2.94)
Control group	34	0 (0.00)	1 (2.94)	1 (2.94)	1 (2.94)	3 (8.82)
x^2^	-					1.063
P	-					0.303

## DISCUSSION

Neonatal upper gastrointestinal hemorrhage is mostly caused by stress ulcers induced by various acute and critical diseases.^8^ Modern medicine has discovered that the gastrointestinal mucosa of newborns is delicate and vulnerable due to the influence of physical development.^9^-^11^ When the body is traumatic, including shock and septicemia, there will be a series of neuroendocrine compensatory responses, sympathetic excitability and increased catecholamine release, thus contracting gastrointestinal vascular smooth muscle, reducing mucosal blood flow, decreasing mucus secretion and increasing gastrin and pepsin secretion.^12^,^13^ Additionally, histamine and acetylcholine stimulate H2 receptor on mucosal parietal cells, thereby increasing H+ secretion, causing mucosal metabolic disorders, and inducing mucosal erosion and even bleeding.^14^,^15^ Without timely intervention, it will not only affect the normal intake of newborns, but also threaten life safety. Consequently, it is of great value to strengthen early bleeding control and restore enteral nutrition for promoting neonatal rehabilitation.

Vitamin K_1_ and ethamsylate are commonly used in the treatment of neonatal coagulation dysfunction.^16^-^17^ Vitamin K_1_ is an essential substance for hepatic synthesis factors II, VII, IX and X, and its mechanism is mainly to promote the transformation of prothrombin precursor into prothrombin, so as to achieve hemostasis.^18^ Moreover, modern pharmacology has found that vitamin K_1_ can also participate in oxidation in the body, increase intestinal peristalsis and secretion.^19^ Ethamsylate is a procoagulant drug, which can effectively reduce capillary permeability, promote vasoconstriction and shorten bleeding time. Furthermore, ethamsylate can also enhance platelet adhesion and shorten coagulation time.

In this study, it was found that the hemostasis time, time of occult blood turning negative and hospital stay of the treatment group were shorter than those of the control group. In addition, the analysis of their stress responses showed that SOD and MDA were more significantly improved in the treatment group, and its total effective rate was higher than that of the control group. The causes may be that norepinephrine combined with cimetidine can reduce body stress responses and protect the gastrointestinal mucosa. Norepinephrine is an α and β adrenergic receptor agonist. Compared with epinephrine hydrochloride, it mainly acts on α receptor and has a strong vasoconstrictive and pressor effect. Injected through a nasogastric tube, norepinephrine can directly contact the hemorrhagic focus and strongly contract capillaries, arterioles and venules, thus improving the overall efficacy.^19^ Cimetidine is a specific competitive H2-receptor antagonist, which plays an important role in inhibiting gastric acid secretion. Moreover, a recent study has found that cimetidine can also inhibit gastric acid secretion caused by histamine, insulin and food stimulation, reduce the acidity of gastric acid, protect the mucosa and prevent upper gastrointestinal hemorrhage.^20^

### Limitations of this study:

There are still some shortcomings in this study. The number of subjects included in this study was limited, so the conclusions drawn may not be very convincing. In addition, we only analyzed and discussed the cases included in our hospital, which may not be representative enough. We look forward to a multi-center study in the future to reach more comprehensive conclusions.

## CONCLUSION

In conclusion, norepinephrine combined with cimetidine in the treatment of neonatal upper gastrointestinal hemorrhage presents high efficacy and safety, so it is worthy of application.

### Authors’ Contributions:

**XD &** **TZ:** Designed this study, prepared this manuscript, are responsible and accountable for the accuracy and integrity of the work.

**HL:** Collected and analyzed clinical data.

**HL** **&** **TZ:** Data analysis**,** significantly revised this manuscript.
